# Enhanced adsorption capacity of ZIF-8 for chemical warfare agent simulants caused by its morphology and surface charge

**DOI:** 10.1038/s41598-023-39507-6

**Published:** 2023-07-28

**Authors:** Sojin Oh, Sujeong Lee, Gihyun Lee, Moonhyun Oh

**Affiliations:** Department of Chemistry, Yonsei University, 50 Yonsei-Ro, Seodaemun-Gu, Seoul, 03722 Republic of Korea

**Keywords:** Metal-organic frameworks, Materials chemistry

## Abstract

The effective separation of toxic chemicals, including chemical warfare agents (CWAs), from the environment via adsorption is of great importance because such chemicals pose a significant threat to humans and ecosystems. To this end, the development of effective porous adsorbents for CWA removal has received significant attention. Understanding the specific interactions between adsorbents and CWAs must precede for the development of effective adsorbents. Herein, we report the relationship between the adsorption capacity of porous ZIF-8 and its morphological and surface characteristics. Four types of ZIF-8, which have different morphologies (such as cubic, rhombic dodecahedron, and leaf- and plate-shaped samples), were selectively prepared. The four types of ZIF-8 were found to have different surface charges owing to dissimilarly exposed components on the surfaces and additionally incorporated components. The specific surface charges of ZIF-8 were found to be closely related to their adsorption capacities for CWA simulants such as 2-chloroethyl ethyl sulfide (CEES) and dimethyl methyl phosphonate (DMMP). Cubic ZIF-8, with the most positive surface charge among four ZIF-8 samples, exhibited the highest adsorption capacity for CEES and DMMP via the effective polar interaction. Moreover, ZIF-8 exhibited excellent recyclability without losing its adsorption capacity and without critical morphological or structural changes.

## Introduction

Chemical warfare agents (CWAs) are highly toxic substances that cause serious long-term damage to humans^1–17^. Isopropyl methylphosphonofluoridate, known as sarin and GB, is an extremely toxic G-type organophosphorus nerve agent that inhibits acetylcholinesterase and causes muscle contraction and asphyxiation through chemical and physical interactions with substrates^1–7^. Bis-(2-chloroethyl) sulfide, known as sulfur mustard and HD, is a blistering agent that damages the exposed skin and tissue^6–8^. Despite the strong will of the international community to protect humans from highly dangerous CWAs, the use of CWAs in military activities, armed conflicts, or terrorist attacks is still occurring, and strategies to mitigate their hazardous effects must be developed. In this context, the effective adsorption, removal, and detoxification of CWAs are of great importance^1–17^. In particular, adsorptive porous materials for efficient adsorption of CWAs must be urgently developed toward human safety. Currently, porous carbons, zeolites, and metal–organic frameworks (MOFs) have shown great potential for the effective adsorption of CWAs^3–15^. Simulants with functionalities similar to CWAs, but with less toxicity and thus convenient to handle in the laboratory, such as 2-chloroethyl ethyl sulfide (CEES) and dimethyl methyl phosphonate (DMMP), are considered as CWA substitutes for this research.

Among several porous materials, MOFs are relatively beneficial because they have several attractive properties such as high surface areas, well-defined pores, versatile structures, and tunable components. MOFs are currently used in many practical applications, such as gas storage, separation, adsorption, catalysis, and sensing^4–12, 16–33^. Numerous studies have been conducted to absorb or separate targeted molecules^18–25^, including CWA simulants using MOFs^4–12^. In addition, several studies have been conducted that the surface charge or morphology of MOFs is an important factor in adsorbing the targeted molecules^34–39^. Among the many MOFs, ZIF-8 is a highly applicable MOF owing to its robust porosity and high thermal and chemical stabilities^40^. Herein, we report the adsorption capacities of porous ZIF-8 toward two critical CWA simulants (CEES and DMMP), depending on the morphological features and specific surface charges of ZIF-8. Four types of ZIF-8 with different morphologies (cubic, rhombic dodecahedron, and leaf- and plate-shaped samples) were selectively synthesized. We found that the four types of ZIF-8 with different morphologies also had different surface charges due to dissimilarly exposed components on the surfaces and additionally incorporated components. In general, ZIF-8 showed excellent adsorption capacities for CEES and DMMP compared to other porous materials. In particular, cubic ZIF-8, with the highest positive surface charge among the four ZIF-8 samples, exhibited the highest adsorption capacities for both CEES and DMMP because of the effective polar interaction of cubic ZIF-8 with the simulants via the electron-rich moieties within the CWA simulants. In addition, the excellent recyclability of ZIF-8 for CEES adsorption was verified, without critical morphological and structural changes.

## Results and discussion

**Figure 1:**
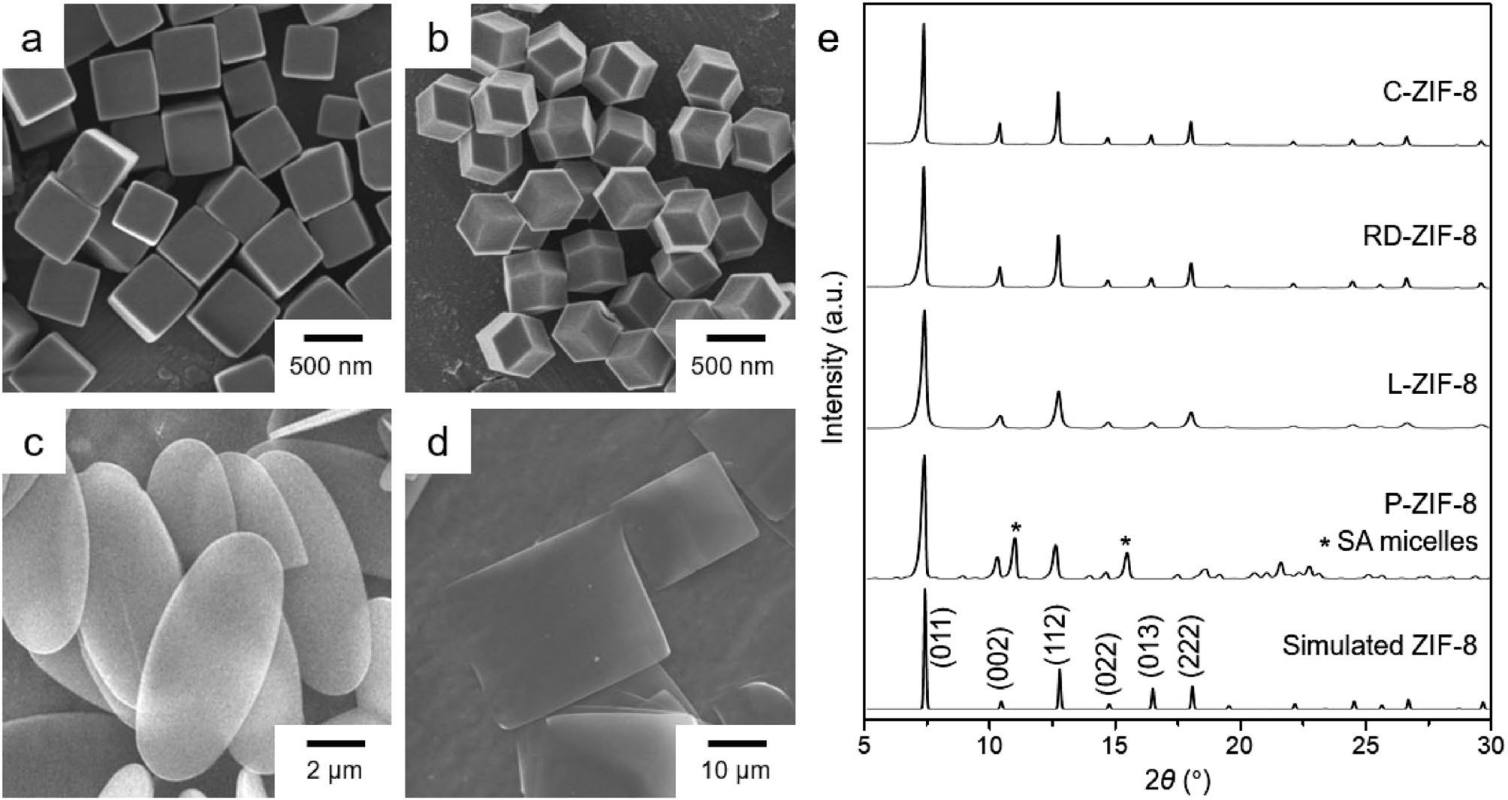
SEM images of (**a**) C-ZIF-8, (**b**) RD-ZIF-8, (**c**) L-ZIF-8, and (**d**) P-ZIF-8. (**e**) PXRD patterns of the four types of ZIF-8 samples having different morphologies and the simulated PXRD pattern of ZIF-8.

First, four types of ZIF-8 with different morphologies were prepared using the reported synthetic methods (see “Experimental” for details). Cubic ZIF-8 (denoted as C-ZIF-8) with exposed {100} planes was prepared by the reaction of Zn(NO_3_)_2_ and 2-methylimidazole (HMeIm) using the reported method^41^. Rhombic dodecahedral ZIF-8 (denoted as RD-ZIF-8) with exposed {110} planes were also synthesized using the reported method^41^. Scanning electron microscopy (SEM) images of the resulting products clearly revealed the formation of uniform cubes and rhombic dodecahedrons of ZIF-8 (Fig. 1a,b). In addition, powder X-ray diffraction (PXRD) patterns show the characteristic peaks of the well-crystalline ZIF-8 materials (Fig. 1e). Energy-dispersive X-ray (EDX) spectra of the products also displayed characteristic elements, including zinc, carbon, and nitrogen, for ZIF-8 (Supplementary Fig. S1). Furthermore, leaf-shaped ZIF-8 (denoted as L-ZIF-8) was obtained from a two-step synthetic process (see “Experimental” for details)^42^. Finally, plate-shaped ZIF-8 (denoted as P-ZIF-8) was prepared via a reported method in the presence of stearic acid (SA) micelles^43^. The SEM images of the resulting products revealed the formation of thin leaf-shaped ZIF-8 particles (L-ZIF-8, Fig. 1c) and square plates of ZIF-8 (P-ZIF-8, Fig. 1d). The PXRD patterns of these products are representative of ZIF-8 (Fig. 1e). However, additional peaks in the PXRD pattern of P-ZIF-8 were detected because of the presence of SA micelles, which are necessary for the formation of P-ZIF-8. The EDX spectra of L-ZIF-8 and P-ZIF-8 displayed the presence of zinc, carbon, and nitrogen elements (Supplementary Fig. S1).

**Figure 2:**
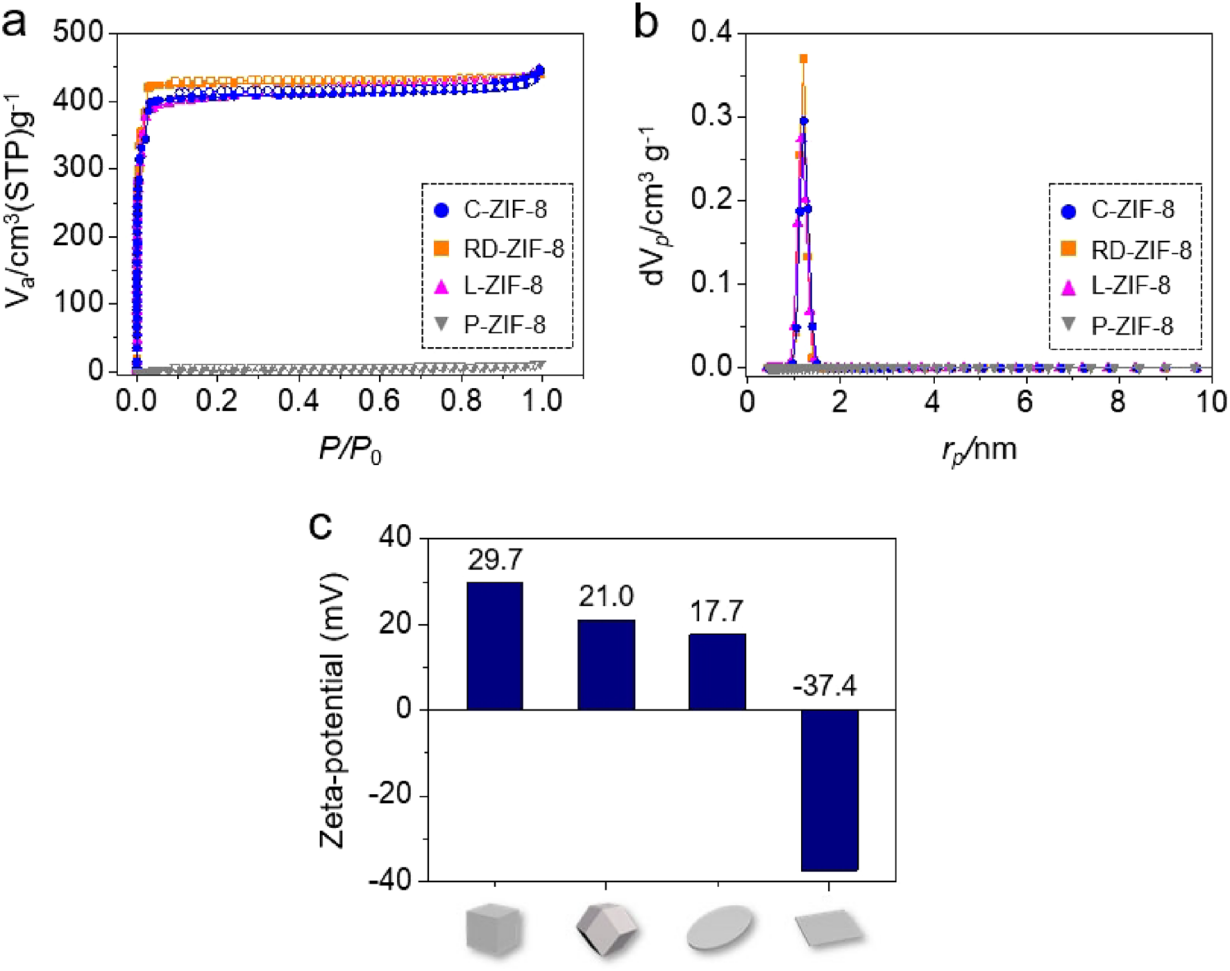
(**a**) N_2_ sorption isotherms of C-ZIF-8 (blue), RD-ZIF-8 (orange), L-ZIF-8 (pink), and P-ZIF-8 (gray). The filled and open symbols represent the adsorption and desorption branches, respectively. (**b**) Pore size distributions of C-ZIF-8 (blue), RD-ZIF-8 (orange), L-ZIF-8 (pink), and P-ZIF-8 (gray) calculated by the NLDFT method. (**c**) Zeta-potentials of the four types of ZIF-8 samples.

The porous properties of the four ZIF-8 samples were analyzed via their N_2_ sorption isotherms at 77 K (Fig. 2a). C-ZIF-8, RD-ZIF-8, and L-ZIF-8 showed the Type I N_2_ sorption isotherm, which is typical for ZIF-8^40^; however, P-ZIF-8 displayed non-porous characteristics because of the SA micelles incorporated within P-ZIF-8^43^, as shown in Fig. 2a. No significant differences were observed in the Brunauer–Emmett–Teller (BET) surface areas and total pore volumes of C-ZIF-8, RD-ZIF-8, and L-ZIF-8 (Supplementary Table S1). For example, the BET surface area and total pore volume of C-ZIF-8 were found to be 1301.1 m^2^ g^−1^ and 0.68 cm^3^ g^−1^, respectively. In addition, the pore size distributions of the ZIF-8 samples determined by the non-local density functional theory (NLDFT) method revealed the characteristic pore dimension of ZIF-8^4, 40^ at ∼ 11.6 Å for C-ZIF-8, RD-ZIF-8, and L-ZIF-8; however, no critical pore was detected in the case of P-ZIF-8 due to the incorporated SA micelles (Fig. 2b). The surface charges of the four ZIF-8 samples were determined from zeta-potential measurements (Fig. 2c). Generally, ZIF-8 is known to have a positive surface charge because of the exposed metal components (Zn^2+^) on the external surface^44, 45^. C-ZIF-8, RD-ZIF-8, and L-ZIF-8 displayed characteristic positive charges; however, they had slightly varied potential values of 29.7, 21.0, and 17.7 mV, respectively (Fig. 2c). Among the four samples, C-ZIF-8 had the most positive surface charge (29.7 mV, possibly due to the presence of many metal components exposed on the surface)^41, 44, 46^. However, the zeta-potential measurement of P-ZIF-8 revealed that it has a negative surface charge (− 37.4 mV, Fig. 2c) because of the co-existing SA micelles. The differences in the surface charges among the four ZIF-8 samples affected their adsorption capacities for the CWA simulants.

**Scheme 1:**
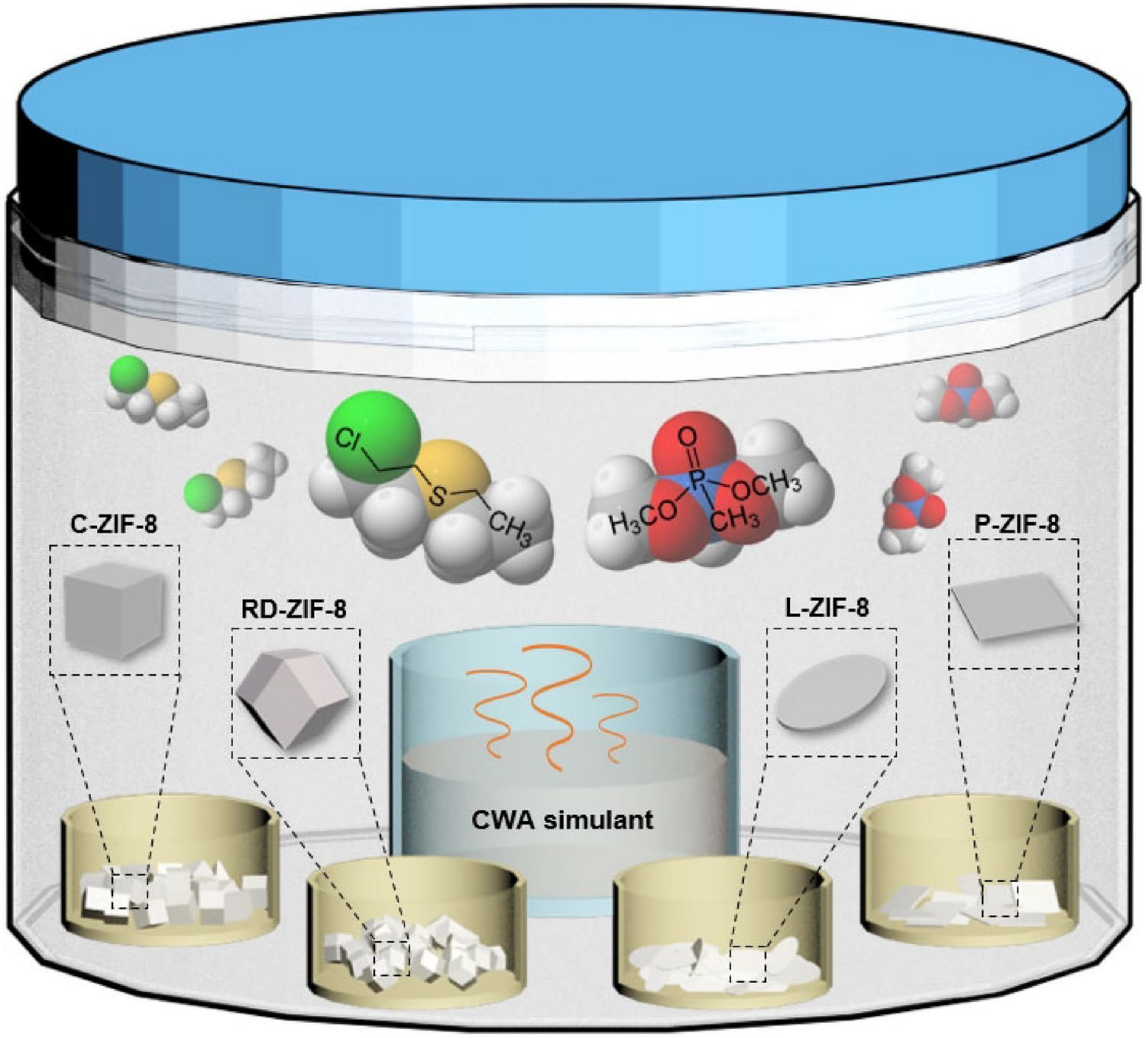
Schematic representation of a jar-in-jar setup for the adsorption experiment of CWA simulants on the four types of ZIF-8 with different morphologies (C-ZIF-8, RD-ZIF-8, L-ZIF-8, and P-ZIF-8).

**Figure 3:**
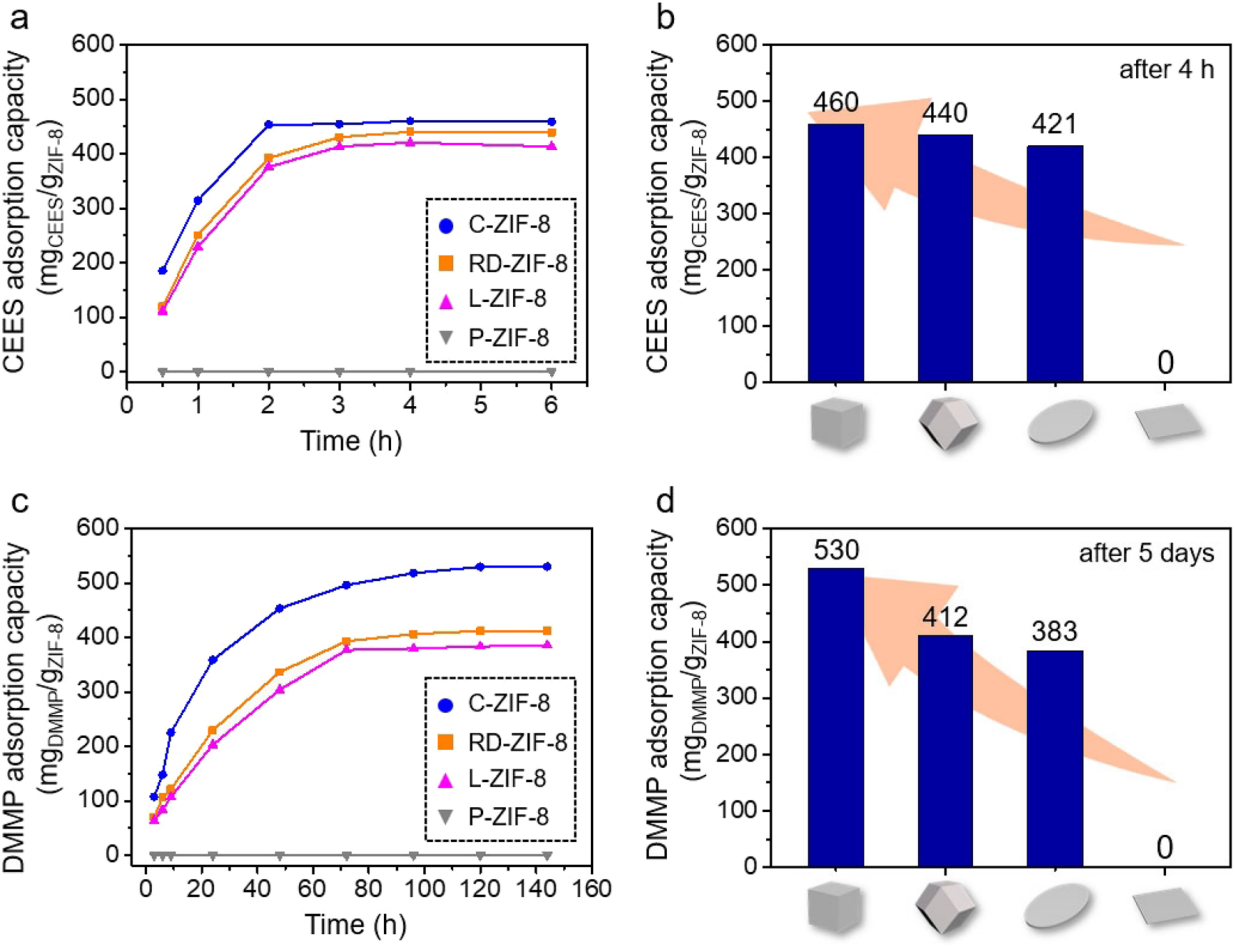
(**a**) Time-dependent CEES adsorption plots for the four types of ZIF-8. (**b**) Adsorption capacities of the four types of ZIF-8 after 4 h exposure to CEES vapors. (**c**) Time-dependent DMMP adsorption plots for the four types of ZIF-8. (**d**) Adsorption capacities of the four types of ZIF-8 after 5 days exposure to DMMP vapors.

**Figure 4:**
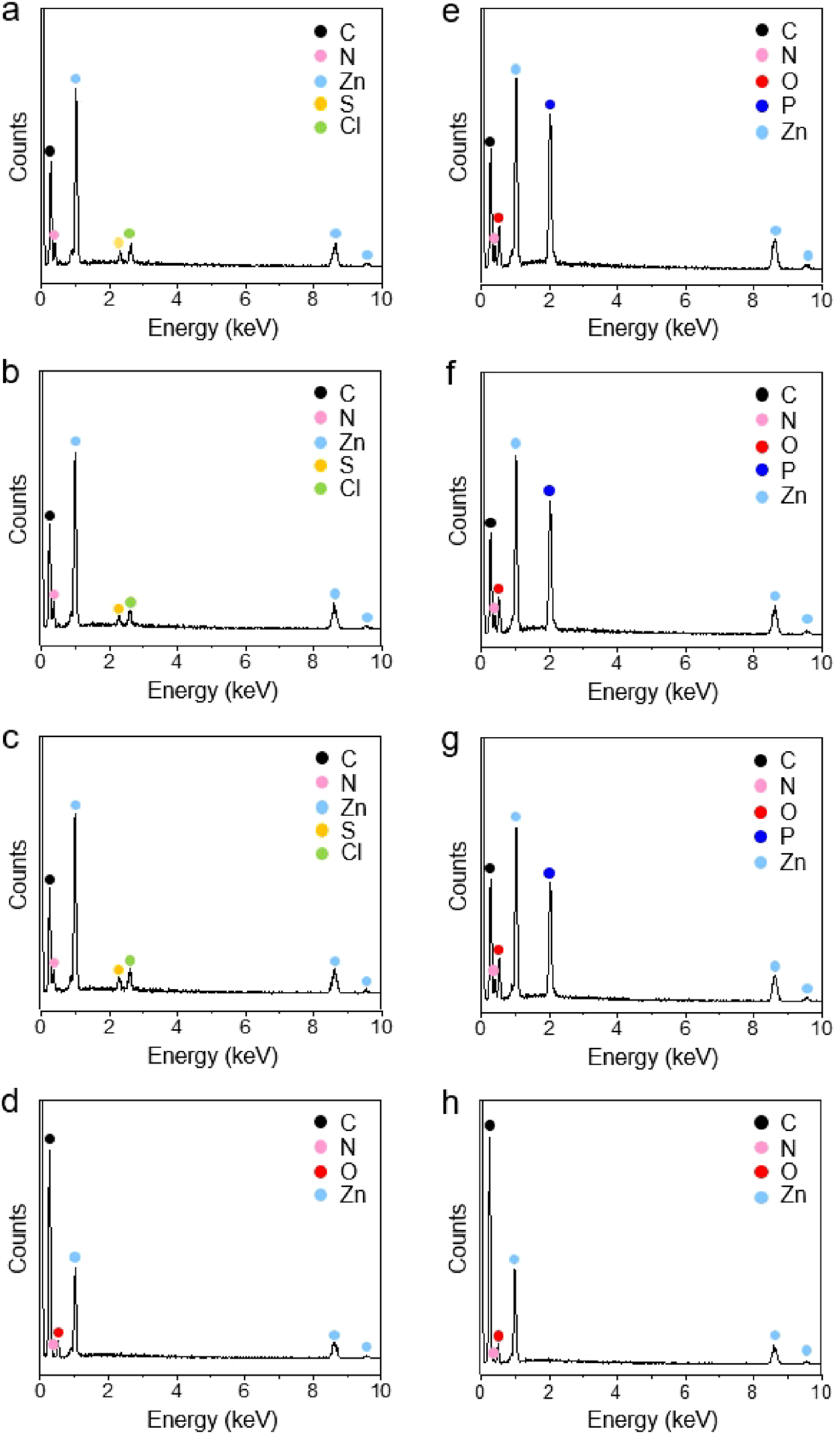
EDX spectra of (**a**) C-ZIF-8, (**b**) RD-ZIF-8, (**c**) L-ZIF-8, and (**d**) P-ZIF-8 after the exposure to CEES vapors. EDX spectra of (**e**) C-ZIF-8, (**f**) RD-ZIF-8, (**g**) L-ZIF-8, and (**h**) P-ZIF-8 after the exposure to DMMP vapors.

The adsorption of CEES on the four ZIF-8 samples was first analyzed at room temperature by using a jar-in-jar setup^4^ (Scheme 1). Small jars containing ZIF-8 samples and CEES were placed together in a large jar, and ZIF-8 samples exposed to CEES vapors for various time periods were analyzed by ^1^H NMR spectroscopy to quantify the uptake amounts of CEES on the ZIF-8 samples. The ZIF-8 samples exposed to CEES vapors for various periods were digested in a mixed deuterated solvent of CDCl_3_ and acetic acid-*d*_4_. Peak integrations of CEES and HMeIm molecules were used to determine the amount of CEES per gram of ZIF-8 (Supplementary Figs. S2–S4). The adsorption graphs showing the relationship between the exposure time and uptake amounts of CEES for the four ZIF-8 samples are shown in Fig. 3a. No significant CEES adsorption was observed on P-ZIF-8, expectedly because of its non-porous nature. The adsorption of CEES on C-ZIF-8, RD-ZIF-8, and L-ZIF-8 almost saturated within 4 h. Moreover, the adsorption capacities of the abovementioned three ZIF-8 samples were slightly different; the adsorption capacity of C-ZIF-8 was found to be the highest at 460 mg of CEES per gram of ZIF-8 (460 mg/g). This adsorption capacity of C-ZIF-8 was much higher than those of other porous materials, such as carbon (74 mg/g)^13^ and zeolite (109 mg/g)^14^ (Supplementary Table S2). The adsorption capacities of RD-ZIF-8 and L-ZIF-8 were 440 and 421 mg/g, respectively (Fig. 3b). The difference in the adsorption capacities of the three ZIF-8 samples can be attributed to their different surface charges; the positive charge of the ZIF-8 samples seems to improve their effective interaction with CEES. The positive charge of ZIF-8 enhances the effective polar interaction with the electron-rich sulfur atoms in CEES^7, 15, 47^. For an effective reaction in adsorption or catalysis, the targeted molecules must be adsorbed on the active site^48–50^. In this case, sulfur atom of CEES can interact with the Lewis acid of Zn^2+^ site by donating a lone pair of electrons^7, 15, 47, 51^. As a result, electron-rich sulfur atoms can be absorbed at the Zn^2+^ site, and this phenomenon is the best in the case of C-ZIF-8, which has the most positive charge due to the exposure of Zn^2+^ on the surface. The adsorption of CEES on the ZIF-8 samples was also verified through IR spectroscopy; the spectra show the representative bands^7, 52^ for CEES at 1262.2 and 1213.0 cm^−1^ (Supplementary Fig. S5). The EDX spectra of the ZIF-8 samples except P-ZIF-8 confirmed the incorporation of CEES into ZIF-8, as shown by the detection of sulfur and chlorine elements (Fig. 4). In addition, the SEM images and PXRD patterns of the ZIF-8 samples after the exposure to and adsorption of CEES revealed no critical morphological and structural changes (Supplementary Figs. S6 and S7).

Furthermore, the DMMP adsorption properties of the four ZIF-8 samples were analyzed by measuring the uptake amounts of DMMP at several time points. The ZIF-8 samples exposed to DMMP vapors for several time points were digested in a mixed deuterated solvent; next, the peak integrations of DMMP and HMeIm molecules were used to determine the amounts of DMMP in the ZIF-8 samples (Supplementary Figs. S8–S10). The adsorption graphs showing the uptake amounts of DMMP for the four ZIF-8 samples are shown in Fig. 3c. No adsorption of DMMP was observed in the case of P-ZIF-8, similar to that of CEES, owing to its non-porous nature. The adsorption of DMMP on the other three ZIF-8 samples was saturated after 5 days. The time required for saturation of DMMP adsorption was much longer than that of CEES adsorption (4 h) because of the lower vapor pressure of DMMP. The vapor pressures of DMMP and CEES at 25 °C are 0.96 and 3.4 mmHg^53, 54^, respectively. The adsorption capacities of the three ZIF-8 samples were slightly different, and the DMMP adsorption capacity of C-ZIF-8 was the highest at 530 mg of DMMP per gram of ZIF-8 (530 mg/g). This value is considerably higher than those of other porous materials, including porous carbon and other MOFs^3, 7, 10^ (Supplementary Table S3). The DMMP adsorption capacities of RD-ZIF-8 and L-ZIF-8 were found to be 412 and 383 mg/g (Fig. 3d). The IR spectra and EDX spectra of the ZIF-8 samples except P-ZIF-8 confirmed the effective adsorption of DMMP on the ZIF-8 samples (Supplementary Figs. S11 and 4). There were no significant morphological or structural changes after DMMP adsorption, as shown in the SEM images and PXRD patterns (Supplementary Figs. S12 and S13).

**Figure 5:**
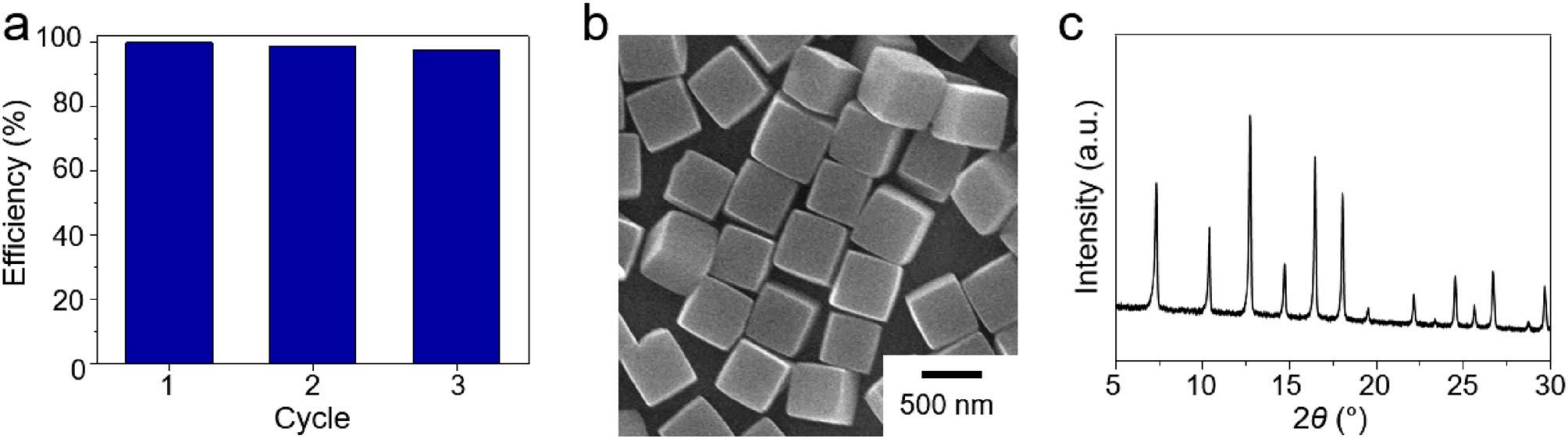
(**a**) Recyclability of C-ZIF-8 over three successive CEES adsorption cycles. Second and third cycles were conducted after removal of the adsorbed CEES via the immersion of the samples in methanol. (**b**) SEM image and (**c**) PXRD pattern of C-ZIF-8 after three successive adsorption cycles.

In addition, the recyclability of C-ZIF-8 for CEES adsorption was tested by conducting three successive adsorption experiments (Fig. 5a). The CEES adsorption capacity of C-ZIF-8 was well preserved during the three cycles. In addition, the SEM image and PXRD pattern (Fig. 5b,c) of C-ZIF-8 after three cycles revealed no critical morphological or structural changes during the adsorption process.

## Conclusion

In conclusion, the effective adsorption of two vital CWA simulants (CEES and DMMP) on porous and stable ZIF-8 was demonstrated. Four types of ZIF-8 (C-ZIF-8, RD-ZIF-8, L-ZIF-8, and P-ZIF-8) with different morphologies and specific surface charges were selectively prepared and were found to exhibit excellent adsorption properties for CEES and DMMP. In particular, C-ZIF-8, which had the highest positive surface charge, exhibited the highest adsorption capacities for both CEES and DMMP. The positively charged surface of C-ZIF-8 effectively attracted polar CEES and DMMP molecules, thus resulting in the highest adsorption capacity. Moreover, the adsorption capacity of C-ZIF-8 was well maintained during three adsorption cycles, confirming the excellent recyclability of C-ZIF-8 during simulant adsorption.

## Experimental

### General methods

All solvents and chemicals were purchased from commercial sources and used as received, unless otherwise stated. Deionized water was obtained from Millipore Direct-Q^®^3. SEM images were obtained using JEOL JSM-7001F field-emission SEM (Yonsei Center for Research Facilities, Yonsei University) and Carl Zeiss SIGMA 55VP field-emission SEM (National Instrumentation Centre for Environmental Management, Seoul National University). EDX spectra were acquired using a Hitachi SU 1510 SEM device equipped with a Horiba EMAX Energy E-250 EDX system. PXRD patterns were obtained using a Rigaku Ultima IV instrument equipped with a graphite monochromated Cu Kα radiation source (40 kV, 40 mA). IR spectra of the solid samples and liquid CWA simulants were acquired using a Jasco FT/IR 4200 spectrometer and the attenuated total reflection module. The adsorption–desorption isotherms of N_2_ (77 K) were measured using a BELSORP Max volumetric adsorption equipment system. All isotherms were measured after pretreatment under dynamic vacuum at room temperature for 3 h. Zeta-potential measurements were carried out in an aqueous solution using a Malvern Nano-ZS Zetasizer. ^1^H NMR spectra were recorded on a Bruker Avance III HD 300 spectrometer (^1^H NMR, 300 MHz), with chemical shifts reported relative to the residual deuterated solvent peaks.

### Preparation of C-ZIF-8

Zn(NO_3_)_2_·6H_2_O (0.1 mmol, 29.7 mg), 2-methylimidazole (HMeIm) (5.5 mmol, 451.6 mg), and hexadecyltrimethylammonium bromide (0.0014 mmol, 0.5 mg) were dissolved in 8 mL of deionized water^41^. The resulting aqueous solution was then incubated at room temperature for 20 min. The product generated within this time was isolated and subsequently washed several times with deionized water and methanol via a centrifugation–redispersion cycle and dried in vacuum for 1 h.

### Preparation of RD-ZIF-8

Zn(NO_3_)_2_·6H_2_O (0.8 mmol, 238.0 mg) and HMeIm (1.7 mmol, 135.6 mg) were dissolved in methanol (30 mL). The resulting solution was then placed in an oil bath at 70 °C for 30 min. The product generated within this time was isolated and subsequently washed several times with methanol via a centrifugation–redispersion cycle and dried in vacuum for 1 h.

### Preparation of L-ZIF-8

The structural transformation of ZIF-L to L-ZIF-8 was conducted via a simple thermal treatment of leaf-shaped ZIF-L. Leaf-shaped ZIF-L was synthesized according to a previously reported procedure^42^. Zn(NO_3_)_2_·6H_2_O (2 mmol, 595.0 mg) and HMeIm (16 mmol, 1313.6 mg) were dissolved in deionized water (80 mL). The resulting aqueous solution was then stirred at room temperature for 4 h. The product generated within this time was isolated and subsequently washed several times with deionized water via a centrifugation–redispersion cycle and dried in an oven at 70 °C. The ZIF-L particles (25.0 mg) were dispersed in 32 mL of a co-solvent (DMF:ethanol = 3:1 *v*/*v*). The resulting solution was sonicated for 5 min and then heated at 70 °C for 30 h. The resulting product was isolated and subsequently washed several times with ethanol via a centrifugation–redispersion cycle and dried under vacuum for 1 h.

### Preparation of P-ZIF-8

P-ZIF-8 was synthesized according to the reported procedure with a modification^43^. Pluronic F127 (20.0 mg) and stearic acid (SA; 0.05 mmol, 14.0 mg) were dissolved in 2 mL of deionized water, placed in an oil bath at 80 °C for 5 h, and incubated at room temperature for 12 h under the static condition to obtain a stable nanoplate SA micelle solution. The SA micelle solution was further dissolved in 33 mL of deionized water and mixed with sodium dodecylbenzenesulfonate (0.04 mmol, 13.9 mg) for 15 min. Then, Zn(NO_3_)_2_·6H_2_O (0.06 mmol, 17.9 mg) dissolved in 1 mL of deionized water was added to the solution and stirred for 15 min at room temperature. HMeIm (4.0 mmol, 328.4 mg) dissolved in deionized water (2 mL) was subsequently added to the solution and stirred for 1 h at room temperature. The solution was then placed in an oil bath at 80 °C for 12 h. The resulting product was isolated and subsequently washed several times with deionized water and ethanol via a centrifugation–redispersion cycle and dried under vacuum for 1 h.

### Adsorption of CWA simulants on ZIF-8 samples

The adsorption of 2-chloroethyl ethyl sulfide (CEES) and dimethyl methylphosphonate (DMMP) on the four ZIF-8 samples was carried out at room temperature using a jar-in-jar setup. The four ZIF-8 samples (5.0 mg) with different morphologies were placed in a ceramic crucible pan, and a CWA simulant (500 μL) was placed in a 5 mL beaker. Subsequently, a ceramic crucible pan and beaker were placed into the jar, the lid was closed, and the jar was sealed using Teflon tape. After a certain period, the amount of the CWA simulant adsorbed on ZIF-8 was quantified through ^1^H NMR spectroscopy. The ZIF-8 samples were digested in a mixed deuterated solvent of CDCl_3_ and acetic acid-*d*_4_ to obtain the ^1^H NMR spectra.

### Recycling of C-ZIF-8 for CEES adsorption

In the recycling test for CEES adsorption on C-ZIF-8, three adsorption cycles were conducted. After the first adsorption cycle, C-ZIF-8 was washed several times with methanol and dried under vacuum for 30 min. The adsorption process was repeated under the same conditions for the second and third cycles.

## Supplementary Information

Supplementary Information.

## Data Availability

All data generated or analysed during this study are included in this published article and its supplementary information files.
