# Supplementary material for: Enhanced adsorption capacity of ZIF-8 for chemical warfare agent simulants caused by its morphology and surface charge
Source: Sci Rep. 2023 Jul 28;13:12250. doi: 10.1038/s41598-023-39507-6 (PMC10382474; doi:10.1038/s41598-023-39507-6)
Supplement: Supplementary file 1 — Supplementary Information. [file 41598_2023_39507_MOESM1_ESM.docx]

**Supplementary Information**

**Enhanced adsorption capacity of ZIF-8 for chemical warfare agent simulants caused by its morphology and surface charge**

Sojin Oh, Sujeong Lee, Gihyun Lee, Moonhyun Oh*

Department of Chemistry, Yonsei University, 50 Yonsei-ro, Seodaemun-gu, Seoul 03722, Republic of Korea
*Corresponding author.

*E-mail*: [moh@yonsei.ac.kr](mailto:moh@yonsei.ac.kr)


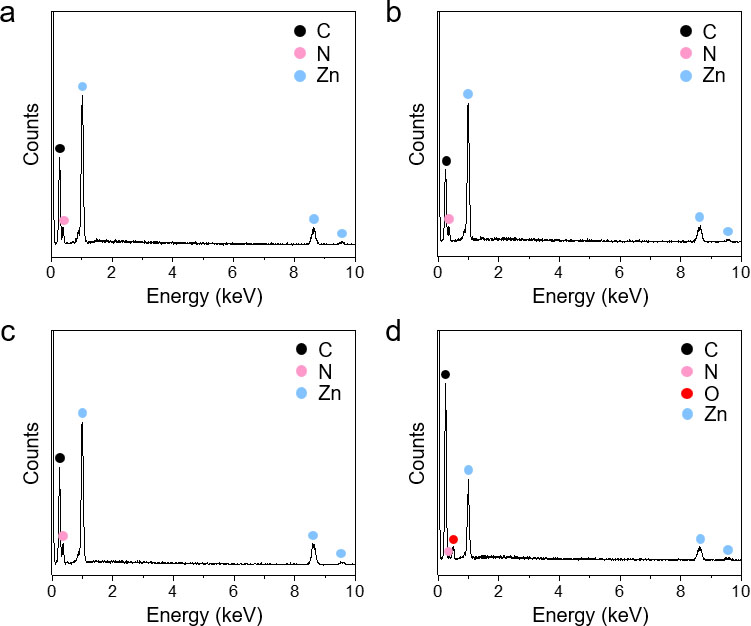


**Figure S1.** EDX spectra of (a) C-ZIF-8, (b) RD-ZIF-8, (c) L-ZIF-8, and (d) P-ZIF-8.


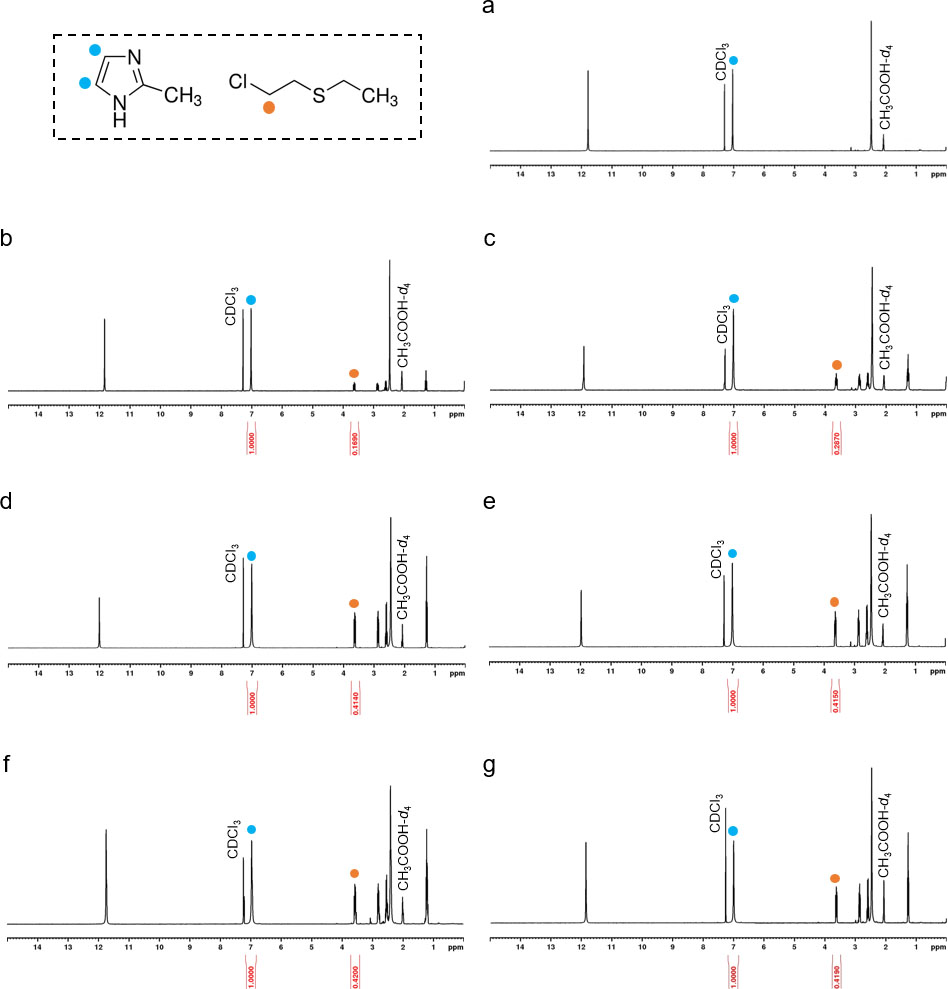


**Figure S2.** ^1^H NMR spectra showing CEES adsorption on C-ZIF-8. ^1^H NMR spectra of (a) pure C-ZIF-8 and C-ZIF-8 exposed to CEES vapors for (b) 0.5, (c) 1, (d) 2, (e) 3, (f) 4, and (g) 6 h, respectively.


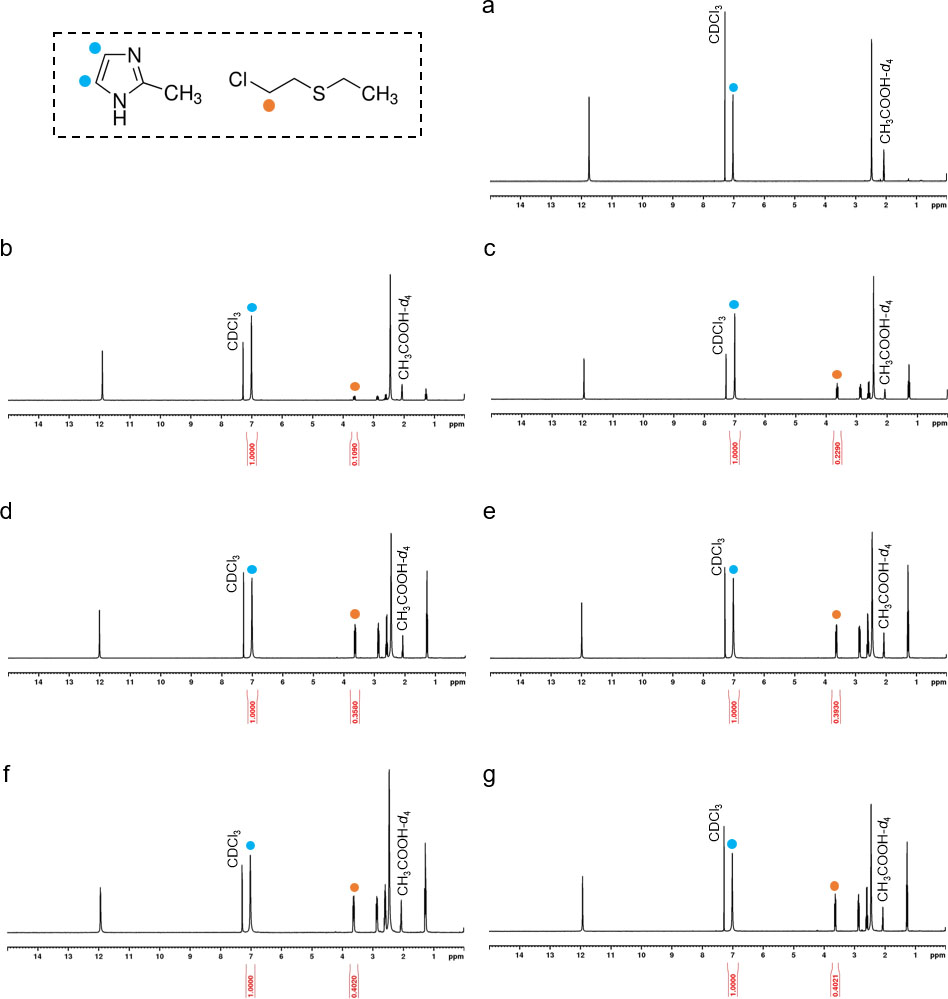


**Figure S3.** ^1^H NMR spectra showing CEES adsorption on RD-ZIF-8. ^1^H NMR spectra of (a) pure RD-ZIF-8, and RD-ZIF-8 exposed to CEES vapors for (b) 0.5, (c) 1, (d) 2, (e) 3, (f) 4, and (g) 6 h, respectively.


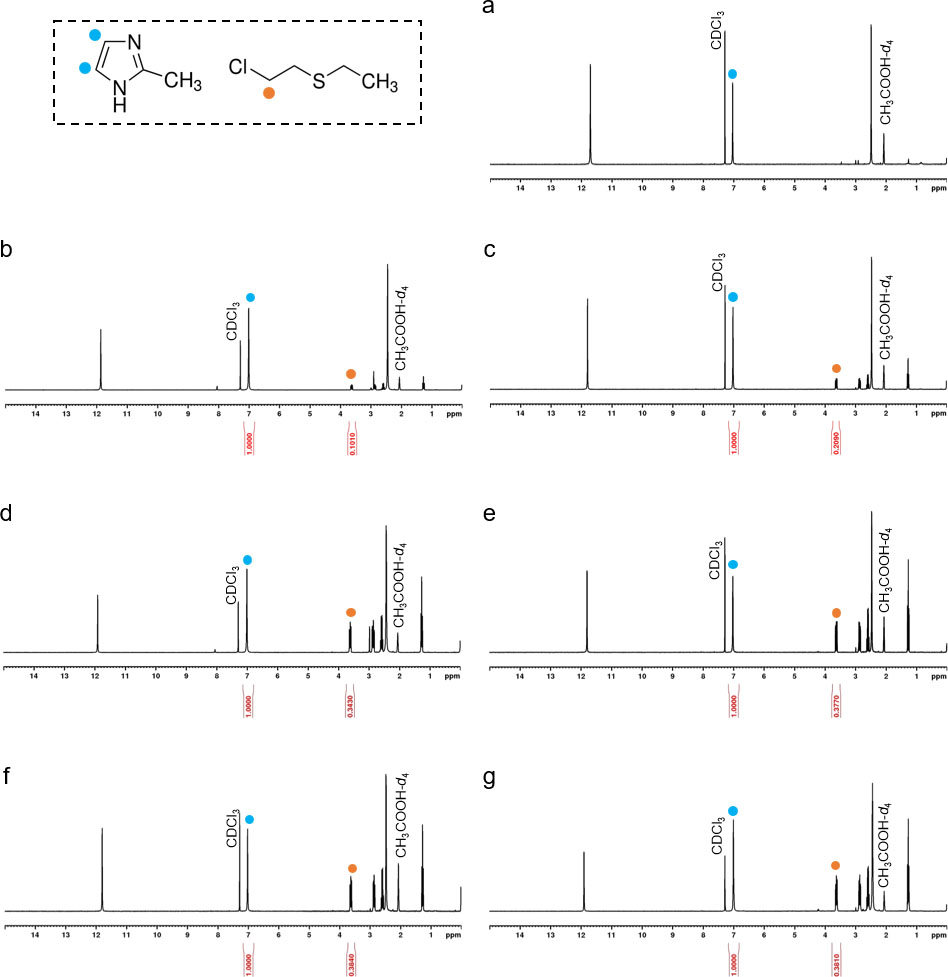


**Figure S4.** ^1^H NMR spectra showing CEES adsorption on L-ZIF-8. ^1^H NMR spectra of (a) pure L-ZIF-8, and L-ZIF-8 exposed to CEES vapors for (b) 0.5, (c) 1, (d) 2, (e) 3, (f) 4, and (g) 6 h, respectively.


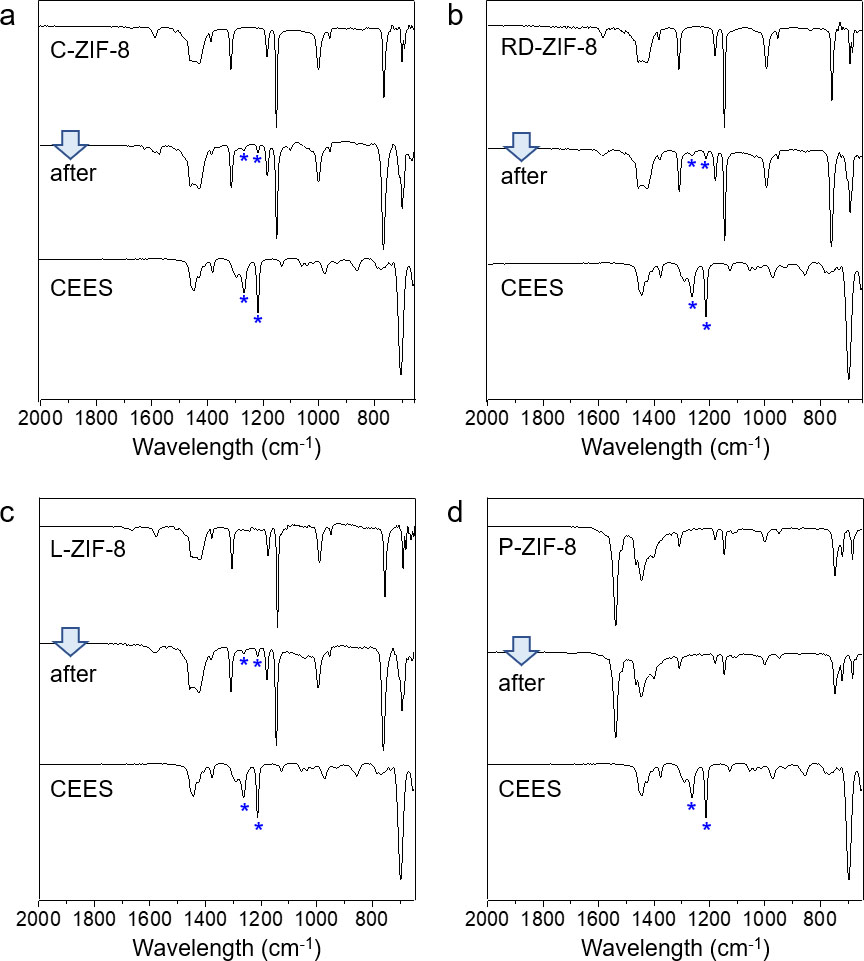


**Figure S5.** IR spectra of (a) C-ZIF-8, (b) RD-ZIF-8, (c) L-ZIF-8, and (d) P-ZIF-8 before and after the exposure to CEES vapors.


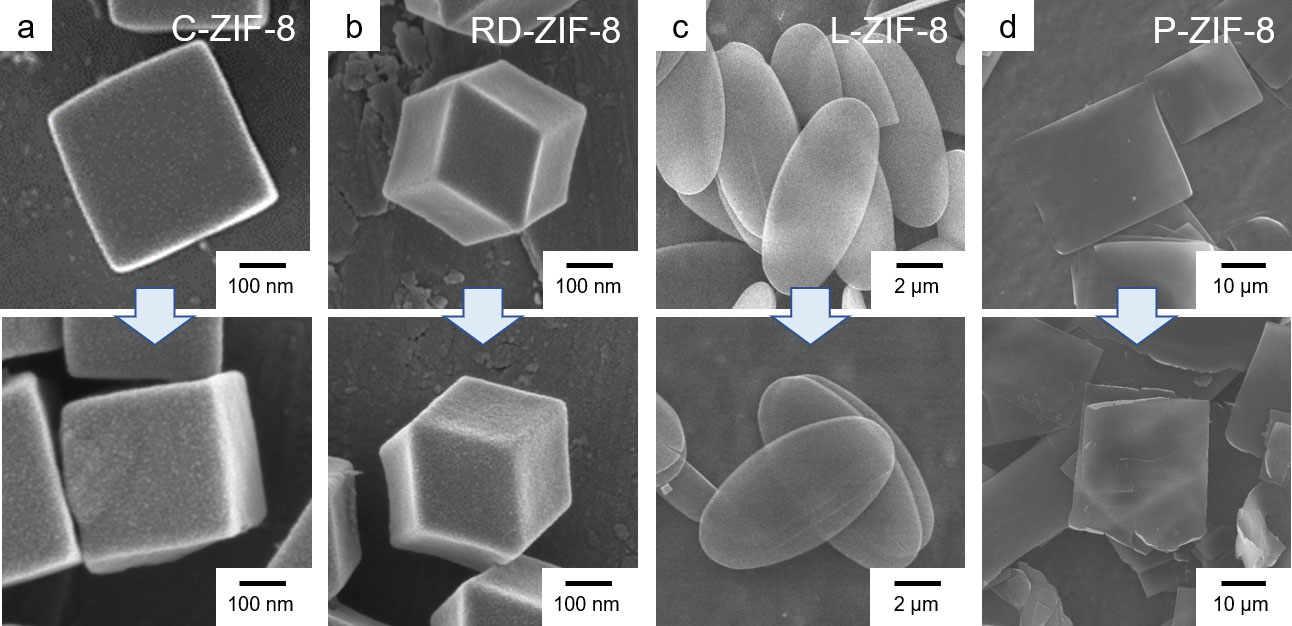


**Figure S6.** SEM images of (a) C-ZIF-8, (b) RD-ZIF-8, (c) L-ZIF-8, and (d) P-ZIF-8 before and after the exposure to CEES vapors.

**
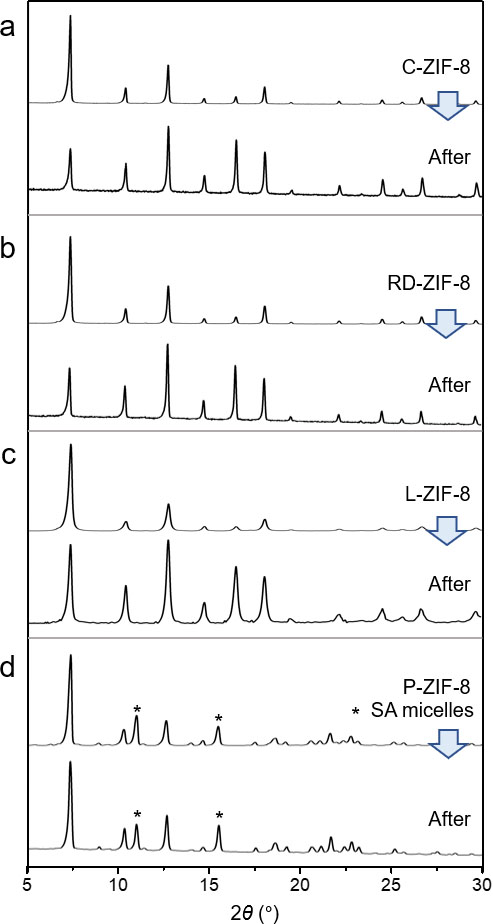
**

**Figure S7.** PXRD patterns of (a) C-ZIF-8, (b) RD-ZIF-8, (c) L-ZIF-8, and (d) P-ZIF-8 before and after the exposure to CEES vapors.

**.**
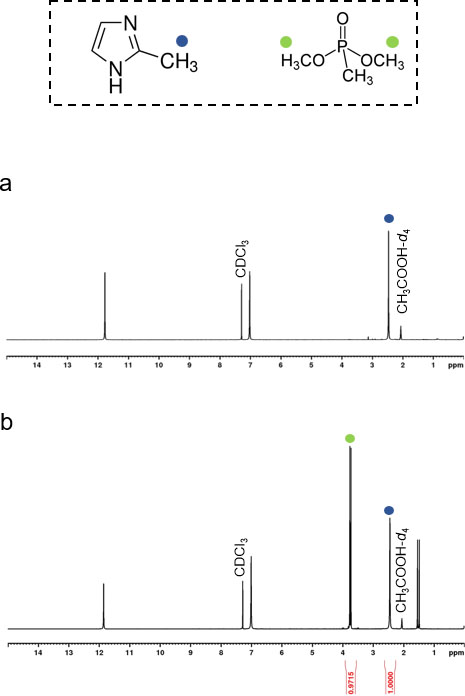


**Figure S8.** ^1^H NMR spectra showing DMMP adsorption on C-ZIF-8. ^1^H NMR spectra of (a) pure C-ZIF-8 and (b) C-ZIF-8 exposed to DMMP vapors for 5 days.


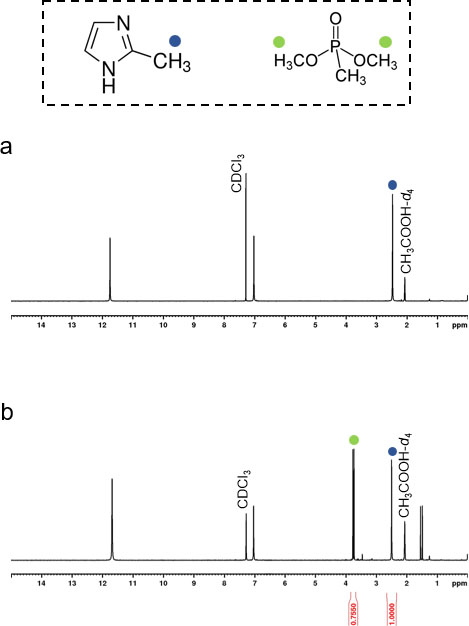


**Figure S9.** ^1^H NMR spectra showing DMMP adsorption on RD-ZIF-8. ^1^H NMR spectra of (a) pure RD-ZIF-8 and (b) RD-ZIF-8 exposed to DMMP vapors for 5 days.


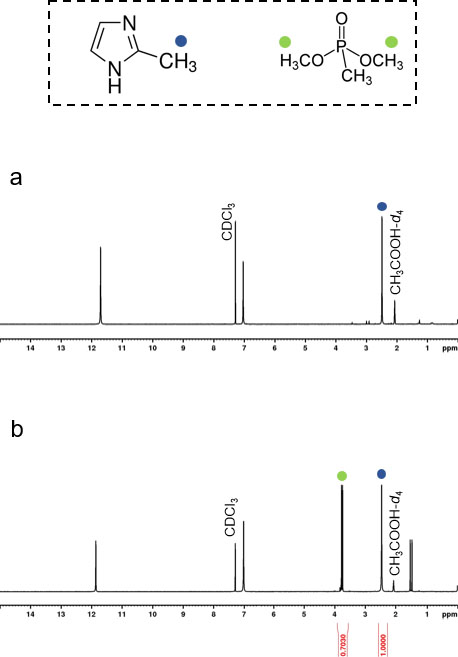


**Figure S10.** ^1^H NMR spectra showing DMMP adsorption on L-ZIF-8. ^1^H NMR spectra of (a) pure L-ZIF-8 and (b) L-ZIF-8 exposed to DMMP vapors for 5 days.


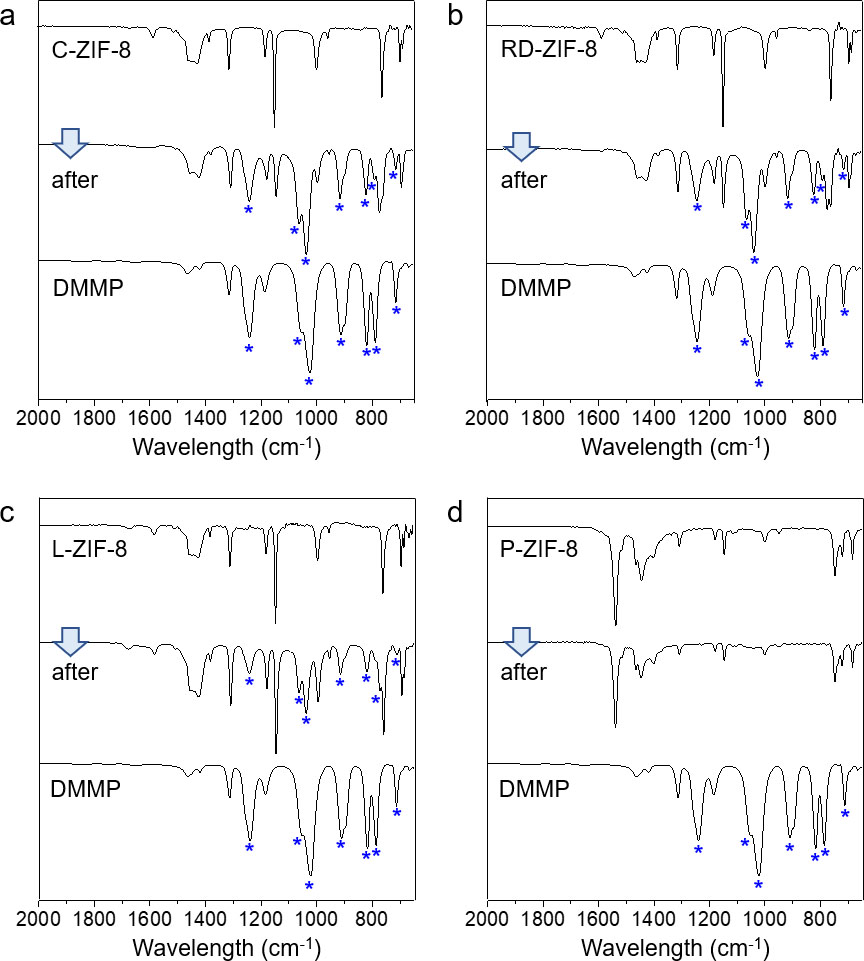


**Figure S11.** IR spectra of (a) C-ZIF-8, (b) RD-ZIF-8, (c) L-ZIF-8, and (d) P-ZIF-8 before and after the exposure to DMMP vapors.


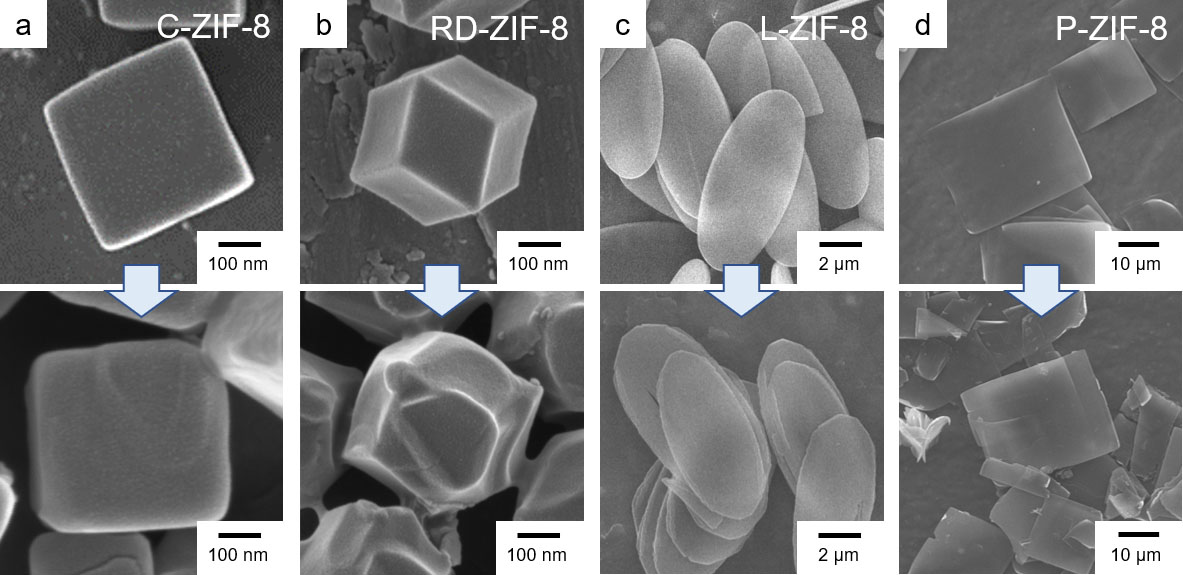


**Figure S12.** SEM images of (a) C-ZIF-8, (b) RD-ZIF-8, (c) L-ZIF-8, and (d) P-ZIF-8 before and after the exposure to DMMP vapors.


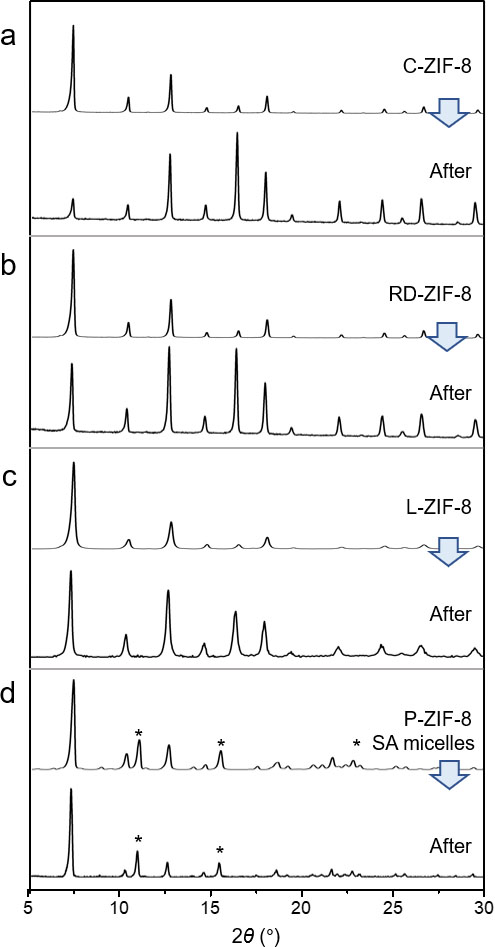


**Figure S13.** PXRD patterns of (a) C-ZIF-8, (b) RD-ZIF-8, (c) L-ZIF-8, and (d) P-ZIF-8 before and after the exposure to DMMP vapors.


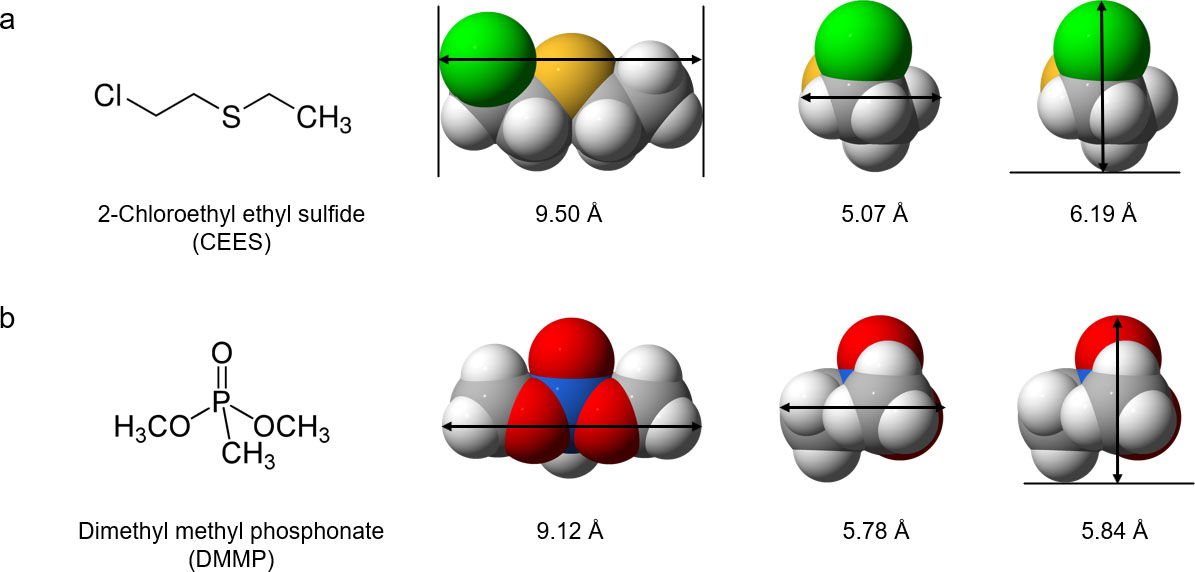


**Figure S14.** Structures and van der Waals models of (a) CEES and (b) DMMP. C: gray, S: yellow, Cl: green, H: white, O: red, P: blue.

**Table S1.** BET surface areas and total pore volumes of C-ZIF-8, RD-ZIF-8, L-ZIF-8, and P-ZIF-8.

|  | Surface area  (m^2^ g^-1^) | Total pore volume (cm^3^ g^-1^) |
| --- | --- | --- |
| C-ZIF-8 | 1301.1 | 0.68 |
| RD-ZIF-8 | 1365.0 | 0.68 |
| L-ZIF-8 | 1335.8 | 0.69 |
| P-ZIF-8 | 9.2 | 0.02 |

**Table S2.** Comparison of adsorption capacities of ZIF-8 and other porous adsorbents for the CEES adsorption.

| Adsorbents | Phase of CEES | Saturation time (h) | Capacity  (mg g^-1^) | Reference |
| --- | --- | --- | --- | --- |
| NU-1000 | Liquid phase | 3.5 | 523 | 1 |
| ZIF-67 | Liquid phase | 0.08 | 463 | 2 |
| CF-CuO | Gas phase  (jar-in-jar setup) | 168 | 74 | 3 |
| (Ag^+^)_55_@Y | Liquid phase | 0.33 | 109 | 4 |
| ZrGO5 | Gas phase  (jar-in-jar setup) | 48 | 205 | 5 |
| C-ZIF-8 | Gas phase  (jar-in-jar setup) | 4 | 460 | This work |
| RD-ZIF-8 | Gas phase  (jar-in-jar setup) | 4 | 440 | This work |
| L-ZIF-8 | Gas phase  (jar-in-jar setup) | 4 | 421 | This work |

**Table S3.** Comparison of adsorption capacities of ZIF-8 and other porous adsorbents for the DMMP adsorption.

| Adsorbents | Phase of DMMP | Saturation time (h) | Capacity  (mg/g) | Reference |
| --- | --- | --- | --- | --- |
| NU-1000 | Liquid phase | 6.5 | 211 | 1 |
| Zr-abtc | Gas phase (breakthrough) | 5.97 | 351 | 6 |
| CMK-8 | Gas phase  (jar-in-jar setup) | 70 | 460 | 7 |
| MgO/C-1 | Gas phase  (breakthrough) | 13.1 | 68 | 8 |
| C-PAC750 (1:2) | Gas phase  (jar-in-jar setup) | 48 | 412 | 9 |
| C-ZIF-8 | Gas phase  (jar-in-jar setup) | 120 | 530 | This work |
| RD-ZIF-8 | Gas phase  (jar-in-jar setup) | 120 | 412 | This work |
| L-ZIF-8 | Gas phase  (jar-in-jar setup) | 120 | 383 | This work |

**References**

1. Asha, P., Sinha, M. & Mandal, S. Effective removal of chemical warfare agent simulants using water stable metal–organic frameworks: mechanistic study and structure–property correlation. *RSC Adv.* **7**, 6691–6696, https://doi.org/10.1039/c6ra28131a (2017).
2. Son, Y.-R., Ryu, S. G. & Kim, H. S. Rapid adsorption and removal of sulfur mustard with zeolitic imidazolate frameworks ZIF-8 and ZIF-67. *Microporous Mesoporous Mater.* **293**, 109819, https://doi.org/10.1016/j.micromeso.2019.109819 (2020).
3. Florent, M., Giannakoudakis, D. A., Wallace, R. & Bandosz, T. J. Carbon textiles modified with copper-based reactive adsorbents as efficient media for detoxification of chemical warfare agents. *ACS Appl. Mater. Interfaces* **9**, 26965–26973, https://doi.org/10.1021/acsami.7b10682 (2017).
4. Son, Y. R., Kim, M.-K., Ryu, S. G. & Kim, H. S. Rapid capture and hydrolysis of a sulfur mustard gas in silver-ion-exchanged zeolite Y. *ACS Appl. Mater. Interfaces* **10**, 40651–40660, https://doi.org/10.1021/acsami.8b15362 (2018).
5. Giannakoudakis, D. A., Mitchell, J. K. & Bandosz, T. J. Reactive adsorption of mustard gas surrogate on zirconium (hydr)oxide/graphite oxide composites: the role of surface and chemical features. *J. Mater. Chem. A* **4**, 1008–1019, https://doi.org/10.1039/c5ta09234e (2016)
6. Chitale, S. K. *et al.* Adsorptive degradation of dimethyl methylphosphonate over Zr-based metal–organic framework built from 3,3′,5,5′-azobenzenetetracarboxylic acid. *J. Hazard. Mater. Lett.* **3** 100066, https://doi.org/10.1016/j.hazl.2022.100066 (2022).
7. Huynh, K. *et al.* Dimethyl methylphosphonate adsorption capacities and desorption energies on ordered mesoporous carbons. *ACS Appl. Mater. Interfaces* **9**, 40638–40644, https://doi.org/10.1021/acsami.7b12033 (2017).
8. Vu, A.-T., Ho, Keon. & Lee, C.-H. Removal of gaseous sulfur and phosphorus compounds by carbon-coated porous magnesium oxide composites. *Chem. Eng. J.* **283**, 1234–1243, https://doi.org/10.1016/j.cej.2015.08.083 (2016).
9. Yu, H. *et al.* Chitosan-derived porous activated carbon for the removal of the chemical warfare agent simulant dimethyl methylphosphonate. *Nanomaterials* **9**, 1703, https://doi.org/10.3390/nano9121703 (2019).
